# Pharmacokinetics of *p*-Aminohippuric Acid and Inulin in Rabbits with Aristolochic Acid Nephropathy

**DOI:** 10.1155/2011/204501

**Published:** 2011-06-16

**Authors:** Chiao-Shih Tseng, Shih-Ming Chen, Shu-Chen Chien, Kuang-Yang Hsu

**Affiliations:** College of Pharmacy, Taipei Medical University, 250 Wu-Hsing Street, Taipei 11014, Taiwan

## Abstract

The characteristics of aristolochic acid nephropathy (AAN) are interstitial fibrosis and atrophy of the proximal tubules, but with no change in glomeruli. To investigate the effects of AA on renal functions and the pharmacokinetics (PKs) of *p*-aminohippuric acid (PAH) and inulin, New Zealand white rabbits were used in this study. The plasma concentrations of PAH and inulin were determined by validated HPLC methods. After a single intravenous administration of 0.5 mg/kg aristolochic acid sodium (AANa), rabbits exhibited mild to moderate nephrotoxicity on the 7th day. Significant tubulointerstitial damage to kidney specimens was found, but there were no remarkable glomerular changes. Clearance rates of PAH and inulin both significantly decreased in AANa-treated rabbits. In addition, there was a significant correlation among the degree of tubulointerstitial changes and PK parameters of PAH after AANa administration, but no correlation was noted with the PKs of inulin. With mild to moderate AAN in rabbits, the renal plasma flow significantly decreased by 55%, and the glomerular filtration rate also significantly decreased by 85%. In conclusion, major renal lesions were found on proximal tubules after AANa administration. The PKs of PAH and inulin significantly changed, and kidney functions, including the RPF and GFR, were reduced.

## 1. Introduction

Chinese herbs inducing nephrotoxicity were first reported in early 1992 by Vanherweghem et al. [1], consisting of two similar cases of rapidly progressive fibrosing interstitial nephritis in young Belgian women who had followed the same slimming regimen. The same herbal name found in the slimming formulas (*Stephanie tetrandra*) had inadvertently been replaced with *Aristolochia fangchi* [2]. Aristolochic acids (AAs), the main compounds of species of *Aristolochia* [3], have been shown to cause tumor induction and renal toxicity in experimental and clinical studies [3–5]. Thus, this renal disease is more appropriately called aristolochic acid nephropathy (AAN). In Belgium [6], more than 100 patients with AAN were recorded, among which 30% had moderate renal failure and 70% were treated by maintenance dialysis or renal grafting, and similar cases were also observed throughout the world [7–12]. In 2001, the US Food and Drug Administration (FDA) advised consumers to discontinue using herbal products which contain AAs [13]. Despite the actions of the FDA and regulatory agencies in other countries, these products containing AAs and suspected to contain AAs are still available on the internet [14].

Morphological features of AAN include extensive hypocellular interstitial fibrosis with atrophy and loss of tubules and are predominantly located in the superficial cortex [15, 16]. However, it is interesting to note that the glomerular structure is not affected [15, 16]. The kidneys play a key role in eliminating xenobiotics. Factors that affect the ability of the kidneys to eliminate drugs may cause marked changes in the pharmacokinetics (PKs) of compounds. In a previous study, renal morphological findings, including damage to the proximal tubules in rabbits after AANa treatment [17], were similar to other experimental studies and reports from human patients [5, 15, 16, 18, 19]. Renal lesions increase as the AANa dose increases, and AAI and AAII show nonlinear PK properties [17]. To investigate the effects of AA on the renal function of rabbits, the current study was performed to assess the PKs of *p*-aminohippuric acid (PAH) and inulin in 0.5 mg/kg AANa-treated rabbits. The renal plasma flow (RPF) and the glomerular filtration rate (GFR) were also investigated.

## 2. Materials and Methods

### 2.1. Chemical Compounds

All chemicals were reagent grade, and all solvents were HPLC grade. Aristolochic acid sodium (AANa, containing 41% AAI and 56% AAII),* p*-aminobenzoic acid potassium salt (≥99%), and *p*-aminohippuric acid sodium salt were obtained from Sigma. Inulin was obtained from Fluka.

### 2.2. Animal Study

The general physiology of rabbits is similar to that of humans [19]. In addition, histopathological features of the tissues of AAN of rabbits are similar to those of humans. Therefore, male New Zealand white rabbits (2.0~3.0 kg) were used for this study. Before the experiment, rabbits were starved for at least 12 h, but water was given ad libitum. Two groups of rabbits were iv-administrated with 20 mg/kg of PAH (*n* = 10) and inulin (*n* = 10) before and on the 7th day after a single intravenous dose of 0.5 mg/kg AANa administration. The data collected before AANa administration is shown as control groups. Blood samples were collected at 5, 10, 20, 30, 45, 60, and 90 min and 2, 3, 4, and 6 h after dosing. After blood sampling, rabbits were sacrificed to obtain kidney specimens. In addition, 3 more rabbits without AANa administration were used as control group for histological examination. All animal experiments conformed to institutional guidelines.

### 2.3. High-Performance Liquid Chromatography (HPLC)

The HPLC instrument consisted of a pump (Shimadzu LD-10AD), UV-VIS detector (Shimadzu-10A), autoinjector (Shimadzu SIL-9A), and integrator (Shimadzu C-R7A). A Cosmosil 5C-18AR (5 *μ*m, 4.6 × 250 mm) column was used to analyze PAH and inulin in plasma samples. The flow rate was set to 1.2 mL/min, and the UV wavelength was set to 290 nm. The mobile phase for PAH consisted of 4% acetonitrile and 0.3% tetramethylammonium chloride in a 0.01 M phosphate butter solution (pH 4.0). The mobile phase for inulin was composed of 2.5% acetonitrile and 0.3% tetramethylammonium chloride in a 0.01 M phosphate butter solution (pH 2.9).

### 2.4. Quantitation of the Plasma Concentrations of PAH

One hundred microliters of 1 *μ*g/mL *p*-aminobenzoic acid (PABA) used as the internal standard with a 4.2% perchloric acid solution was added to 200 *μ*L of a plasma sample and then mixed and centrifuged at 13,000 rpm for 5 min. The supernatant was transferred to another tube and then supplemented with 200 *μ*L dichloromethane. The tubes were vortexed and centrifuged at 13,000 rpm for 5 min again, and the supernatant was transferred to another tube which contained 200 mg ammonium sulfate and 200 *μ*L ethyl acetate. The tubes were vortexed and centrifuged at 13,000 rpm for 5 min once again, and then the supernatant was transferred to another tube. After desiccation under nitrogen gas, the sample was reconstituted with 200 *μ*L of the mobile phase, and 50 *μ*L was used for HPLC. The concentration range of the standard curve was 0.1~30 *μ*g/mL. This analytical method was validated according to the “Guidance of Bioanalytical Method Validation.” [20] The respective retention times of PAH and PABA were 4.4 and 10.3 min, respectively, with no significant endogenous peak being coeluted in the corresponding chromatograms. The linear regression equation of the standard curve was *Y* = 0.2615*X* + 0.0064 with a correlation coefficient of 0.9999. The limit of quantitation of PAH was 0.1 *μ*g/mL.

### 2.5. Quantitation of the Plasma Concentrations of Inulin

One hundred microliters of 1 *μ*g/mL PABA as the internal standard with 7% perchloric acid solution was added to 200 *μ*L of a plasma sample and then mixed and centrifuged at 13,000 rpm for 5 min. The supernatant was transferred to another tube and then supplemented with 200 *μ*L dichloromethane. After vortexing and centrifuging at 13,000 rpm for 5 min, this supernatant was transferred to another tube and left in boiling water for 30 min to hydrolyze inulin to fructose and allow the fructose to be converted to 5-(hydroxymethyl)-2-furaldehyde (HMF). Then the samples were cooled in cold water for 5 min and transferred to another tube containing 200 mg ammonium sulfate and 200 *μ*L ethyl acetate. The tubes were vortexed and centrifuged at 13,000 rpm for 5 min again. The supernatant was transferred to another tube. After desiccation under nitrogen gas, the sample was reconstituted with 200 *μ*L of the mobile phase, and 50 *μ*L was used for HPLC. The concentration range of the standard curve was 0.1~30 *μ*g/mL. This analytical method was validated according to the “Guidance of Bioanalytical Method Validation.” [20] The respective retention times of inulin and PABA were 8.6 and 11.7 min with no significant endogenous peak being coeluted in the corresponding chromatograms. The linear regression equation of the standard curve was *Y* = 0.1282*X* − 0.1386 with a correlation coefficient of 0.9995. The limit of quantitation of inulin was 5 *μ*g/mL.

### 2.6. Data Analysis and Statistics

The plasma concentration-time data of PAH and inulin of the rabbits were entered into the computer program, PKCALC [21], to obtain the initial estimated parameters, and these were individually weighted with another computer program, WinNonlin [22]. All of the pharmacokinetic parameters, including the distribution half-life (*α*-*t*_1/2_), elimination half-life (*β*-*t*_1/2_), clearance (CL), volume of the distribution at steady state (*V*ss), volume of the distribution (*V*), volume of the distribution in the central compartment (*Vc*), elimination rate constant of the central compartment (*k*_10_), and the transfer rate constant between the central and peripheral compartments (*k*_12_, *k*_21_), were directly obtained from the WinNonlin output. The area under the curve (AUC) was determined using the trapezoidal rule with the area from blood sampling time to infinity estimated as the last drug concentration divided by the estimated terminal elimination rate constant, *k*. This was calculated by dividing 0.693 by *β*-*t*_1/2_.

RPF was calculated using the PAH extraction ratio (*E*_PAH_) which was obtained from Gouyon and Guignard [23] values of 92.9% ± 1.9% observed in 29 normoxemic rabbits and of 90.0% ± 2.7% observed in 21 hypoxemic rabbits were used. The other renal function parameters were calculated with the following equations: filtration fraction = GFR/RPF, GFR = CL_inulin_, RPF = CL_PAH_/*E*_PAH_, and net secretion of PAH = CL_inulin_ − CL_PAH_ [24, 25].

All data are expressed as the mean ± SE. Statistical significance was determined by two-way analysis of variance (ANOVA) for PK parameters.

### 2.7. Histological Examination

Blocks of renal tissues were fixed in 10% buffered formalin for a routine histological examination. Because of minimal changes in the glomeruli, the severity of renal changes was graded by the degree of tubulointerstitial changes. Periodic acid-Schiff- (PAS-) stained sections were evaluated at a magnification of 100×, and findings for the cortex were semiquantitatively scored. The tubulointerstitial histological score was determined as described by Sato et al. [18]. The scores used to evaluate changes in the epithelial tubules were defined as follows: 0, no degeneration of the tubular epithelium; 1, one group or a single degenerated tubule; 2, several clusters of degenerated tubules; 3, moderate degeneration of the tubular epithelium; 4, more severe degeneration of the tubular epithelium; 5, very severe degeneration of the tubular epithelium, with massive necrosis and atrophy present. Mononuclear cell infiltration into the interstitium was evaluated by the following four-point scale: 0, absent; 1, a few scattered cells; 2, groups of mononuclear cells; 3, dense and widespread infiltration. The existence of hyaline cylinders in distal tubules was evaluated as follows: 0, absent and 1, cylinders present. Interstitial fibrosis was defined as follows: 0, absent; 1, mildly diffuse fibrosis; 2, moderate fibrosis; 3, severe fibrosis. The histological score of each rabbit was expressed as the sum of these 4 scores. Nonparametric variables were analyzed by the Mann-Whitney *U*-test. Statistically significant differences between groups were defined at *P* < .05.

## 3. Results

### 3.1. Pharmacokinetics of PAH

**Figure 1:**
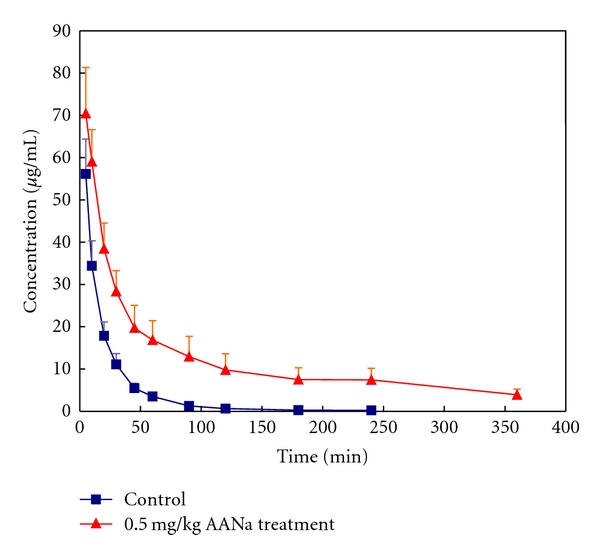
Plasma concentrations of *p*-aminohippuric acid (PAH) after iv administration of 0.5 mg/kg aristolochic acid sodium (AANa) to rabbits (*n* = 10).

The plasma concentration-time curves of PAH after a single iv-administrated dose of 0.5 mg/kg AANa are shown in Figure 1. The PK data before and after AANa administration are given in Table 1. It was observed that after AA administration, CL (*P* = .010) and *k*_10_  (*P* < .001) were reduced by half. *α*-*t*_1/2_, *β*-*t*_1/2_, and AUC significantly increased (*P* < .05). RPF was calculated using *E*_PAH_ which was obtained from the work by Gouyon and Guignard [23]. In the control group, the *E*_PAH_ was 92.9% and RPF was 22.36 ± 4.01 mL/kg/min. Thus, in the group administrated AANa, the extraction ratio was 90.0% and the RPF was 10.11 ± 2.72 mL/kg/min, which significantly differed from control rabbits (*P* < .05).

### 3.2. Pharmacokinetics of Inulin

**Figure 2:**
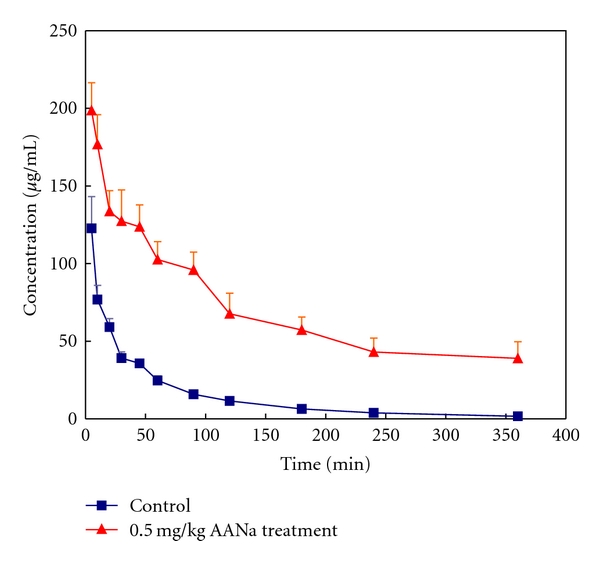
Plasma concentrations of inulin after iv administration of 0.5 mg/kg aristolochic acid sodium (AANa) to rabbits (*n* = 10).

Table 2 shows the PK data of inulin before and on the 7th day after 0.5 mg/kg of AANa iv administration. The plasma concentration-time curves are shown in Figure 2. The CL and *k*  (*P* < .001) significantly decreased, and the AUC significantly increased. The GFR significantly decreased from 5.95 ± 1.79 to 0.89 ± 0.18 mL/kg/min in the AAN group.

### 3.3. Renal Histological Findings after Administration of AANa

**Figure 3:**
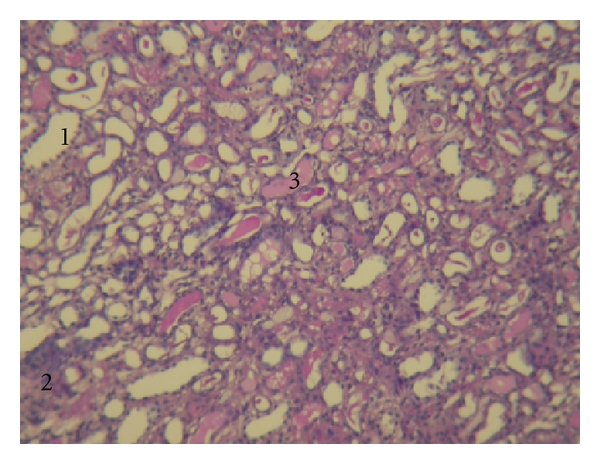
Light microscopic findings of kidney specimens from rabbits after treatment with 0.5 mg/kg aristolochic acid sodium (AANa). The renal histology changed 7 days after 0.5 mg/kg AANa iv administration. (PAS stain, magnification ×100). (1) Degeneration of tubular epithelium; (2) mononuclear cell infiltration; (3) existence of hyaline cylinders.

**Figure 4:**
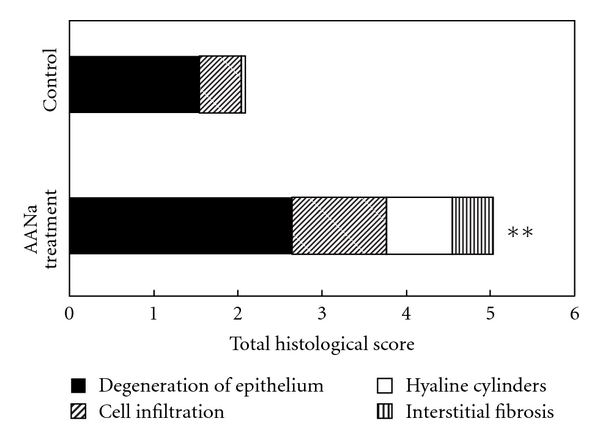
Tubulointerstitial histological scores of rabbits after treatment with 0.5 mg/kg aristolochic acid sodium (AANa). **P < .01.

On the 7th day after a single iv administration of 0.5 mg/kg AANa, rabbits were sacrificed to obtain renal specimens. Moderate proximal tubular damage with 0.5 mg/kg AANa treatment was seen (Figure 3). The histological score in the control included degeneration of tubular epithelium (1.55 ± 0.38), cell infiltration (0.49 ± 0.39), and hyaline cylinders (0.05 ± 0.05), but interstitial fibrosis was absent. However, after AANa treatment on day 7, the degeneration of the tubular epithelium (2.66 ± 0.09, *P* < .01), cell infiltration (1.11 ± 0.10), hyaline cylinders (0.78 ± 0.06, *P* < .05), interstitial fibrosis (0.49 ± 0.11, *P* < .05) were observed. The sums of the histological scores on day 7 were 2.09 ± 0.71 in the control and 5.03 ± 0.23 (*P* < .01) in AANa-treated rabbits (Figure 4). Moreover, compare with the previous study [17], there was no significant difference in histological results of 0.5 mg/kg AANa-treated rabbits. Despite these tubular changes, there was no remarkable glomerular change.

## 4. Discussion

The pathology of AAN [15, 16] is characterized by a poor ability for regeneration and a potent tendency towards fibrosis without glomerular injury. Thus far, renal plasma flow and glomerular filtration changes in AAN have not been evident. AAs can induce nephrotoxicity and tumor toxicity in experimental studies [5, 18, 19], and the toxicity of AAs is irreversible [26, 27]. In the present study, a single iv administration of 0.5 mg/kg AANa was chosen according to a previous study [17]. On day 7 after administration of AANa, the characteristic histological changes in rabbits were mild to moderate degeneration of the tubular epithelium and interstitial fibrosis, and remarkable existence of hyaline cylinders in distal tubules. The overall degree of nephrotoxic effects was mild to moderate, and the sum of the histological score was 5.03 ± 0.23, similar to the findings of a previous study [17]. The experimental models of AAN were induced by repeated doses, the histological findings of the proximal tubules [5, 18, 19] were similar to those of this study, and no alternation was found in glomerular capsules [18, 19].

PAH is cleared by both filtration and secretion, and thus has, been used to estimate RPF and the function of renal anion secretion transporters (OATs) [24, 28]. Both OAT1 and OAT3 [29–31], which mediate the transport of PAH, are located in proximal tubules which exhibit the main damage with AAN. The RPF with 20 mg/kg PAH in control rabbits showed no significant difference between this study (22.36 ± 4.01 mL/kg/min) and one by Guignard et al. (13.66 ± 1.83~25.54 ± 3.14 mL/kg/min) [32–35]. Some studies [36, 37] have reported that reductions in the peritubular capillary (PTC) density and tubulointerstitial fibrosis area increased in AAN kidneys. Therefore, ischemia and hypoxia occur in AAN, and this may contribute to progressive tubulointerstitial fibrosis. Thus, in the group administrated AANa, the extraction ratio was 90.0% and the RPF was 10.11 ± 2.72 mL/kg/min, which significantly differed from those of control rabbits (*P* < .05). The PK data determined from the iv administration of AANa are given in Table 1. When AAN was present, the results showed significant decreases in CL and *k*_10_ and significant increases in *α*-*t*_1/2_ and *β*-*t*_1/2_. Altered pharmacokinetic parameters of PAH in AAN rabbits appear to be linked to several factors: first, the decreased RPF and the diminished delivery of PAH to nephrons or renal tubules (22.36 ± 4.01 versus 10.11 ± 2.72 mL/kg/min, *P* < .05). Many PTCs in AAN animals [36] and patients [37] are distorted and collapsed. PTC injury is conceivably related to a decreased blood supply to renal tubules [38]. Second, the GFR (5.95 ± 1.79 versus 0.89 ± 0.18 mL/kg/min, *P* < .05) and net secretion (14.82 versus 8.21 mL/kg/min) also decrease PAH elimination. Moreover, there was a close correlation between the degree of tubulointerstitial changes and pharmacokinetic data of PAH after AA administration; interstitial infiltration (*r* = 0.79, *P* < .01); and overall tubulointerstitial lesions (*r* = 0.64, *P* < .05) were positively correlated with the AUC, and hyaline cylinders (*r* = 0.73, *P* < .05) and interstitial fibrosis (*r* = 0.72, *P* < .05) were negatively correlated with *β*_1/2_. The net secretion decline influenced by interstitial inflammation and fibrosis in proximal tubules affected PK changes of PAH.

Inulin was used to measure the GRF. No significant difference was seen in GFR at 5.95 ± 1.79 mL/kg/min in control rabbits compared to reports by Guignard et al. [32, 33, 39] and Pichette et al. [40] (3.69 ± 0.37~6.4 ± 0.6 mL/kg/min). In this study, CL significantly decreased by −85% and *k* by −53%, while the AUC increased by 594% after AANa treatment as shown in Table 2. However, there was no correlation between the PK data for inulin in AANa-treated rabbits and the degree of tubulointerstitial lesions. Inulin is eliminated through the renal route only via filtration, and therefore tubulointerstitial lesions had no direct correlation with the PKs of inulin. Although the glomerular structure remained unchanged at the light microscopic level, levels of GFR significantly declined (*P* < .05) in mild to moderate AAN. The abrupt fall in the GFR may be associated with afferent vasoconstriction, transtubular backleak of the filtrate, and interstitial inflammation. The characteristics of AAN are extensive interstitial inflammation and fibrosis in the proximal tubules and increases in renal interstitial pressure obstructing the reabsorption of isoosmotic fluid across the tubular epithelium. The filtration fraction plays a role in determining the tubular isoosmotic reabsorption efficiency, particularly in proximal tubules [41]. In this study, a decreasing trend in the filtration fraction from 27% to 4% represents net water reabsorption and reductions in filtrated solutes, such as sodium chloride and glucose. The early development of normal glycemic glucosuria observed in AAN patients [42] and in these AA-treated animals [5, 19] might explain how the lesions in proximal tubules enhance the passive tubular isoosmotic filtrate backleak, while the filtration fraction decreases, in part, as an indication of reduced proximal tubular sodium reabsorption leading to an increased sodium chloride concentration and delivery to the macula densa. This enhanced tubuloglomerular feedback sensitivity and renin secretion [43] as a consequence of afferent vasoconstriction lead to a fall in the GFR. Furthermore, sodium reabsorption was constantly blocked with the progression of disease, which also influenced water reabsorption in the proximal tubules and then a decrease in the volume of extracellular fluid contraction. This inference proves that a decrease in the distribution volume of inulin was used as a measure of extracellular fluid [44]. Then, a decrease in the extracellular fluid volume causes a decline in the blood volume. The constriction of afferent arterioles may lead to decreases in both the GFR and the RPF through a tubuloglomerular feedback mechanism. However, further studies are needed to investigate these speculations.

In conclusion, mild to moderate nephrotoxicity was seen on day 7 after a single administration of AANa. After AANa administration, major renal lesions were found on proximal tubules. The PKs of PAH and inulin significantly changed, and kidney functions, including the RPF and GFR, were reduced. There was a significant correlation between the degree of tubulointerstitial changes and PK parameters of PAH after AANa administration, but no correlation was noted with the PKs of inulin.

## Figures and Tables

**Table 1:** Effects of 0.5 mg/kg aristolochic acid sodium (AANa) treatment on *p*-aminohippuric acid in rabbits (*n* = 10).

	Control	AANa treatment	*P* value
CL (mL/kg/min)	20.77 ± 3.73	9.10 ± 2.45	.010
*k*_10_ (min ^−1^)	0.091 ± 0.009	0.040 ± 0.009	<.001
*k*_12_ (min ^−1^)	0.053 ± 0.018	0.024 ± 0.005	.104
*k*_21_ (min ^−1^)	0.057 ± 0.008	0.039 ± 0.009	.147
*α*-*t*_1/2_ (min)	5.41 ± 1.10	10.85 ± 2.22	.016
*β*-*t*_1/2_ (min)	25.09 ± 3.25	94.28 ± 28.56	.026
*Vc* (mL/kg)	228.72 ± 30.38	246.09 ± 36.84	.713
*V*ss (mL/kg)	404.26 ± 51.28	509.32 ± 165.07	.587
AUC (*μ*g × min/mL)	1291.49 ± 192.96	4376.83 ± 1123.55	.017

*Data are expressed as the mean ± SE.

CL, clearance; *k*, elimination rate constant; *k*_10_, elimination rate constant of the central compartment; *k*_12_ *_,_* *k*_21_, the transfer rate constant between the central and peripheral compartments; *α*-*t*_1/2_, distribution half-life; *β*-*t*_1/2_, elimination half-life; *V*, volume of the distribution; *Vc*, volume of the distribution in the central compartment; *V*ss, volume of the distribution at steady state; AUC, area under the curve.

**Table 2:** Effects of 0.5 mg/kg aristolochic acid sodium (AANa) treatment on inulin in rabbits (*n* = 10).

	Control	AANa treatment	*P* value
CL (mL/kg/min)	5.95 ± 1.79	0.89 ± 0.18	.014
*k* (min ^−1^)	0.015 ± 0.001	0.007 ± 0.001	<.001
*V* (mL/kg)	452.42 ± 173.38	130.91 ± 10.90	.089
AUC (*μ*g × min/mL)	4644.96 ± 549.68	32246.76 ± 6531.83	.002

*Data are expressed as the mean ± SE.

Parameters are described in the footnotes to Table 1.
